# Posterior Cortical Atrophy: Clinical Presentation and Cognitive Deficits Compared to Alzheimer’s Disease

**DOI:** 10.1155/2005/762569

**Published:** 2005-08-04

**Authors:** Raquel F. Charles, Argye E. Hillis

**Affiliations:** ^1^Department of MedicineJohns Hopkins University School of Medicine1830 E. Monument StreetNinth FloorBaltimoreMD 21287USA; ^2^Department of NeurologyJohns Hopkins University School of MedicinePhipps 126; 600 N. Wolfe StreetBaltimoreMD 21287USA

## Abstract

*Background:* Posterior cortical atrophy (PCA) is an uncommon dementia syndrome with initial manifestations of visual dysfunction and preservation of memory and language until late in the disease. Since prognosis and management differ from typical Alzheimer’s disease (tAD), clinical tests to distinguish PCA from tAD are needed.

*Methods:* Fifteen PCA cases and 15 tAD cases, defined by clinical and MRI criteria, were compared by present symptoms and scores on four cognitive tests.

*Results:* Symptoms of visual disturbance and dyslexia were more commonly reported in PCA cases (*p* = 0.0001 and *p* = 0.006, respectively), and memory loss was more commonly reported in tAD (*p* = 0.006). Patients with PCA were less accurate on the Cortical Vision Screening Test (*t* = 6.0; *p* < 0.001) and in copying the Rey-Osterreith Complex Figure (*t* = 6.0; *p* < 0.001), in comparison to the tAD group. Memory, evaluated by the Rey Auditory Verbal Learning Test, was impaired in both groups; however, delayed recall was more impaired in the tAD group (*t* = 2.5; *p* = 0.03).

*Conclusion:* Compared to patients with tAD, patients with PCA are more likely to present to their providers with symptoms of visual dysfunction. Performance on simple tests of visual perception and copying can be used to distinguish the two disorders even a few years after initial symptoms.

## 1. Introduction

Posterior cortical atrophy (PCA) has been considered a "visual variant of Alzheimer's disease" [23]. However, unlike typical clinically defined Alzheimer's disease (using NINCDS-ADRDA criteria [25]; hereafter tAD), this uncommon dementia syndrome has initial manifestations of visual dysfunction with preservation of memory and language until late in the disease. These findings make it a unique clinical disorder with its own prognosis and management. Although the earliest symptoms distinguish PCA and tAD, almost by definition (visual symptoms in PCA, memory symptoms in tAD), it is less clear whether appropriate testing can distinguish these syndromes from each other and from other dementing illnesses later in the course, when patients commonly present to tertiary care centers or dementia centers. The frequency of PCA relative to tAD is unknown; it is likely higher in tertiary referral centers than in the community, but may be under-diagnosed in the community.

PCA, like AD, affects people of older age with the majority of cases occurring at age 60 or older. Early-onset cases, however, can occur [23,40]. Benson and colleagues were among the first to describe five cases and suggested a separate classification for the disorder [4]. Supported by many case reports, the disorder has been characterized as a progressive dementia syn-

ISSN 0953-4180/05/$17.00 © 2005 – IOS Press and the authors. All rights reserved drome including early visual dysfunction such as visual agnosia, alexia, anomia, transcortical sensory aphasia, environmental agnosia, constructional apraxia, features of Gerstmann's syndrome (agraphia, acalculia, right-left confusion, and finger agnosia) and development of Balint's syndrome. Features of Balint's syndrome include optic ataxia, the inability to move one's hand to an object using vision; ocular apraxia, the inability to voluntarily direct gaze to a particular point; and simultanagnosia, the inability to perceive two or more objects simultaneously [1,3–6,8,10–12,14–16,19,24,26–29,32,33,35,37,39]. PCA has also been classified into two subtypes based on anatomical location of the lesion: dorsal, which presents with Balint's syndrome and apraxia, and ventral, which presents with alexia and visual agnosia. Both classically have preserved memory and insight until late in the illness [1,4,11,14, 16,24,28,37].

The clinical and neurobehavioral findings characteristic of PCA are associated with distinct radiological findings including posterior cortical (posterior parietal and occipital, sometimes asymmetric) atrophy demonstrated on magnetic resonance imaging (MRI) and computed tomography (CT) [1,3–5,11,12,14–16,29,35,36]. Positron emission tomography (PET) and single photon emission computed tomography (SPECT) imaging demonstrate hypometabolism/hypoperfusion in posterior parietal and occipital regions as well [1,6,7,14–16, 26,27,29,32,33,35,36,39].

Although PCA and tAD differ in presentation and radiological findings, the etiology of the two disorders is often identical. Neuropathological studies have revealed an Alzheimer's type pathology in most cases of PCA, with microscopic evidence of neurofibrillary plaques and tangles [2,5,10,15,19,20,24,33,35–37]. However, in patients with PCA the plaques and tangles are predominantly located in the primary visual cortex and visual association areas, and gross atrophy is localized to this posterior cortical area [19,20,24,33]. These findings are consistent with the difference in clinical symptoms between PCA and tAD. It is unknown why in some cases of AD the pathology localizes to the occipital and parietal areas, whereas in most cases the pathology localizes to the temporal and parietal areas. Subcortical gliosis and spongiosis with neuronal loss associated with Creutzfeldt-Jakob Disease have also been described in PCA cases [36]. However, the pathology of corticobasal degeneration and dementia with Lewy bodies, other dementias associated with visuospatial deficits, have not been described [8]. Therefore, the distinction between PCA and tAD is similar to the distinction between different forms of frontotemporal lobar degeneration (FTLD) (nonfluent progressive aphasia, semantic dementia, and the dysexecutive form of frontotemporal dementia). It is also similar to the distinction between these clinical forms of FTLD and corticobasal degeneration or progressive supranuclear palsy, which can all have the same histopathology (but with different distributions in the brain) and even the same genetic mutation [22].

Although there have not yet been controlled studies to evaluate the effectiveness of medications in this syndrome, treatment with medications such as acetyl cholinesterase inhibitors are commonly used, since Alzheimer's type pathology predominates in PCA cases. Occupational therapy to learn compensatory techniques to deal with their visual impairment is also very useful in patients with PCA who suffer from constructional apraxia and impaired visual perception. Their relatively preserved insight and learning allows these patients to benefit more from therapy than patients with tAD. For this reason, quickly identifying patients based on symptoms and neuropsychological tests focusing on visual-perceptual skills will enable these patients to receive the appropriate therapy to cope with this disorder. We hypothesized that presence of visual dysfunction relative to memory dysfunction and impairment on visual perception tests can be used to distinguish PCA from tAD even years after onset of initial symptoms (when patients typically present to dementia centers, questioning a previous diagnosis of stroke, tAD, or Creutzfeldt-Jakob disease).

## 2. Methods

### *2.1. Study design*

The study was approved by the Johns Hopkins Internal Review Board. Fifteen patients with PCA presented to the Johns Hopkins Hospital Cognitive Neurology clinic between 2000 and 2003. Patients received a full clinical examination including neurological examination, screening of medications, laboratory testing, and MRI to rule out other potential causes of their cognitive symptoms, as well as neuropsychological testing performed by trained staff. Patients were required to have visual dysfunction with accompanying occipito-parietal atrophy by MRI and/or hypoperfusion in this region demonstrated on PET or SPECT imaging (see Fig. 1 for examples) to be included in the study. A neurologist specializing in dementia and a neuroradiologist

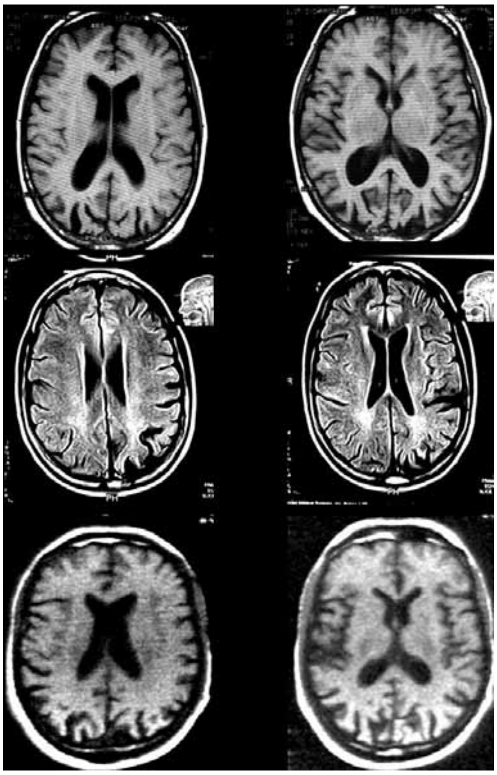

Fig. 1. MRI of PCA patients. Axial MRI images of three patients with PCA, illustrating marked occipital and posterior parietal atrophy.

evaluated all MRI, CT, PET, and/or SPECT imaging scans.

The PCA cases were compared to fifteen cases of tAD who presented to Johns Hopkins Hospital Cognitive Neurology clinic for initial evaluation or follow-up over a 4-month period in 2002–2003. All patients with tAD met criteria for clinically probable AD, as outlined by the National Institutes of Neurological and Communication Disorders and Stroke-Alzheimer's Disease and Related Disorders Association [25]. These patients also received a full clinical examination including neurological examination, screening of medications, laboratory testing, and MRI to rule out other potential causes of their dementia, as well as neuropsychological testing performed by trained staff. Patients with tAD had generalized or specifically temporo-parietal atrophy on MRI and/or temporo-parietal hypoperfusion on SPECT imaging (see Fig. 2 for examples).

Both groups underwent a battery of neuropsychological testing as part of their clinical examination. The battery included the following tests: Weschler Memory Scale III Orientation and Information [38]; The Mini Mental State Examination (MMSE) [13]; Digit Span (forward and backward); the Grooved Pegboard Test [Psychological Assessment Resources, Inc., 1999]; The Trail Making Test [31] Parts A and B; Stroop Color Task and Stroop Color-Word Test [17]; Controlled Oral Word Association [34]; Rey-Osterreith Complex Figure tests of immediate and delayed recall [30]; oral reading of a paragraph; written description of a complex picture; writing the alphabet; drawing a clock; and time perception. Oral reading of the paragraph was scored by number and percentage of words misread and by the number of seconds required to complete the paragraph. In addition, the battery included the following tests that were evaluated as potential candidates for distinguishing PCA from tAD: the Cortical Vision Screening Test [21] (which evaluates visual acuity, shape and size discrimination, shape detection, spatial scanning, perceptual identification, reading skills, face perception, and perception when symbols are closely versus widely spaced); the Boston Naming Test [18] (which evaluates visual recognition and word retrieval); the Rey-Osterreith Complex Figure test of direct copying (which evaluates visuospatial

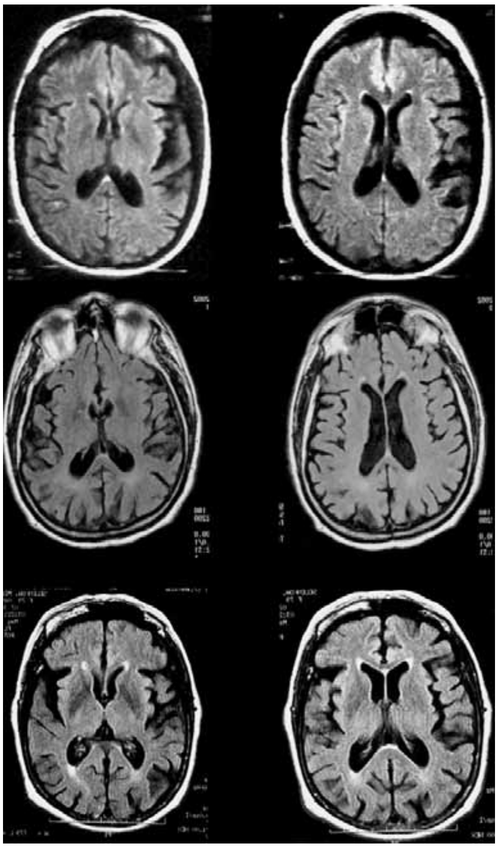

Fig. 2. MRI of AD patients. Axial MRI images of patients with AD, illustrating marked temporoparietal atrophy and constructional skills); and the Rey Auditory Verbal Learning Test (R.A.V.L.T., Western Psychological Services, 1996, which evaluates verbal learning across trials). Symptoms at the time of presentation to that clinic were obtained with a standard questionnaire and interview of the patient and the caregiver, specifically asking about problems with attention, recent and remote memory, reading, writing, driving, word-retrieval, language comprehension, speech production, motor skills, visual perception, and spatial skills (e.g., getting lost).

### 2.2. Statistical analysis

Differences between groups in scores on the Cortical Vision Screening Test, the Boston Naming Test, the Rey-Osterreith Complex Figure, and the Rey Auditory Verbal Learning Test were evaluated using the paired-samples T-test and the Fisher's Exact Test (F.E.), using an alpha level of $p < 0.05$ for a significant difference. Then cut-off scores that distinguished PCA from tAD were identified for those tests that showed a significant difference between groups using the paired-samples T-test. These cut-off scores were evaluated with Fisher's exact analyses, and the sensitivity and specificity of each test for identifying PCA were determined.

## 3. Results

### 3.1. Patient characteristics (*Table 1*)

There were no significant differences between patients with PCA and patients with tAD in terms of age of onset (mean 61.4 ± 7.2 years, compared to 66.5 ± 11.9 years), age at time of testing (mean 65.3 ± 6.6 compared to 69.3 ± 11.7 years), disease duration at time of presentation (mean 4.0 ± 2.3 compared to 3.3 ± 1.4 years), or MMSE scores (a rough estimate of disease severity; mean 20.3 ± 6.1, compared to 18.6 ± 5.0) as shown in Table 1. The proportion with a college education was similar: 67% of the PCA cases and 53% of the tAD cases. Both groups had an equally higher predominance of females at 73%.

### 3.2. Differences in presenting symptoms (*Table 2*)

The most common symptom of patients with PCA at the time of presentation to the clinic was visual dysfunction reported by 60% of the patients, significantly greater than in the AD group, in which no patients reported this complaint (Fisher's exact: $p = 0.001$). Reading impairment was reported in 46.7% of the patients with PCA, and was not reported in any of the tAD cases ($p = 0.006$). Memory loss was also a symptom of PCA cases, reported in 53.3% of patients, but was significantly less frequent than in tAD cases, where memory loss was a reported symptom of all patients ($p = 0.006$). Dysgraphia ($p = 0.48$), dyscalculia ($p = 1.0$), and dysnomia ($p = 1.0$) did not reach the threshold for significance between the two groups.

### 3.3. Differences in neuropsychological tests (*Table 1 and 2*)

Patients with PCA were more impaired than patients with tAD in visual perception, evidenced by lower accuracy on the Cortical Vision Screening Test ($p < 0.001$; see Table 1). Figure copying using the Rey-Osterreith Complex Figure test was also more impaired in the PCA group ($p < 0.001$). In contrast, deficits in verbal learning assessed using the Rey Auditory Verbal Learning Test were more severe in patients with AD than in those with PCA ($p = 0.03$). There was no difference in picture naming, as assessed by scores on the Boston Naming Test. To determine if patients in the two groups made different types of errors on the Boston Naming Test, errors were classified as visual errors (e.g., "apple slices" for escalator), semantic errors (e.g., "calculator" for protractor), visual-semantic errors (e.g., "horse" for unicorn), phonological errors (e.g., "uniform" for unicorn), circumlocutions (e.g., "a mythological animal" for unicorn), or mixed/other (e.g., "tree with a bird in it" for ostrich). There were similar errors types across the two groups, but patients with PCA made a higher proportion of visual errors (27% versus 8% of errors) and semantic/visual-semantic errors (29% versus 23%). Patients with AD made a higher proportion of circumlocutions (39% versus 16% of errors). There was a statistically significant difference between groups in the number of semantic/visual-semantic errors ($p = 0.001$) and the number of circumlocutions ($p = 0.002$). Only patients with AD made phonological errors.

Having identified significant differences between groups on three tests, we identified cut-off scores that seemed to distinguish PCA from tAD cases. On the Cortical Vision Screening Test, 100% of PCA cases and 27.3% of tAD cases scored lower than 88% correct (Fisher's exact: $p = 0.001$). On the Rey Complex Figure (direct copy), all patients with PCA received a score less than 18, while only 13.3% of tAD cases were as impaired ($p < 0.001$). Thus, accuracy below 88%

Table 1
Comparisons of demographics and continuous scores for PCA versus tAD cases (using t-tests)

| | PCA | tAD | t | p value |
|---|---|---|---|---|
| *Patient characteristics* | | | | |
| Mean age of onset (in years) | 61.4 $\pm$ 7.2 | 66.5 $\pm$ 11.9 | $-1.51$ | 0.153 |
| Mean disease duration (in years) | 4.0 $\pm$ 2.3 | 3.3 $\pm$ 1.4 | 1.113 | 0.284 |
| Mean MMSE score | 20.3 $\pm$ 6.1 | 18.6 $\pm$ 5.0 | 0.810 | 0.426 |
| *Neuropsychological tests* (*mean scores*) | | | | |
| CORVIST % error | 34.3 $\pm$ 12.57 | 5.86 $\pm$ 8.28 | 5.95 | $< 0.001$ |
| Rey-Osterreith complex figure score | 7.34 $\pm$ 5.90 | 25.19 $\pm$ 8.10 | $-$ 5.956 | $< 0.001$ |
| RAVLT | | | | |
| Verbal learning | 22.08 $\pm$ 14.68 | 19.38 $\pm$ 9.32 | 0.664 | 0.519 |
| Delayed verbal learning | 2.92 $\pm$ 4.44 | 0.46 $\pm$ 0.97 | 2.45 | 0.030 |
| Boston naming test | 40.56 $\pm$ 17.69 | 33.64 $\pm$ 18.26 | 0.921 | 0.379 |

PCA – Posterior cortical atrophy, tAD – typical Alzheimer's Disease, CORVIST – Cortical Vision Screening Test; RAVLT – Rey Auditory Verbal Learning Test.

Table 2
Comparisons of dichotomous scores and symptoms for PCA versus tAD cases (using fisher exact)

| | PCA | tAD | p value |
|---|---|---|---|
| *Reported symptoms* (%) | | | |
| Visual difficulty | 60 | 0 | 0.001 |
| Dyslexia | 46.7 | 0 | 0.006 |
| Dysgraphia | 13.3 | 0 | 0.48 |
| Dyscalculia | 13.3 | 6.7 | 1.0 |
| Memory loss | 53.3 | 100 | 0.006 |
| Dysnomia | 6.7 | 6.7 | 1.00 |
| *Neuropsychological tests* (%) | | | |
| CORVIST $>$ 12% error | 100 | 27.3 | 0.001 |
| Rey-Osterreith complex figure score $\leqslant$ 18 | 100 | 13.3 | $< 0.001$ |
| Normal verbal learning on RAVLT | 23.1 | 0 | 0.087 |
| Normal delayed verbal learning on RAVLT | 23.1 | 0 | 0.098 |
| Impaired picture naming | 41.7 | 57.1 | 0.69 |

PCA – Posterior cortical atrophy, tAD – typical Alzheimer's Disease, CORVIST – Cortical Vision Screening Test; RAVLT – Rey Auditory Verbal Learning Test.

on the Cortical Vision Screening Test had sensitivity of 100% and specificity of 73% for this group. A score less than 18 on the Rey-Osterreith Complex Figure had sensitivity of 100% and specificity of 87%. The two tests combined ($<$ 88% correct on the Cortical Vision Screen Test and $<$ 18 on the Rey Complex Figure test) had a sensitivity of 100% and specificity of 100%. On the Rey Auditory Verbal Learning Test (delayed recall), 23% of PCA cases had normal scores, whereas no patients with tAD performed within normal range, but this difference was not significant using the Fisher exact test. Normal delayed memory measured by the Rey-OsterreithVerbal Learning Test has a sensitivity of only 23%, but a specificity of 100% for tAD.

## 4. Illustrative cases

The distinct presentation of PCA can be best illustrated with a few cases with different subsets of the initial symptoms characteristic of PCA.

### *4.1. Case 1*

A 59 year-old professional with a graduate education first noticed difficulty reading at the age of 56. Shortly thereafter, personal finances became the responsibility of his wife, because he developed trouble not only with reading, but also with writing and calculation. Driving and topographical memory also became a problem for him. Over the course of a year, his visual deficits progressed to not being able to find what he was looking for or tell which was the head versus the tail of his dog. Instead of relying on his vision, he would use touch to compensate for his visual dysfunction. He sought medical attention from an ophthalmologist regarding his visual problems, who identified incomplete left homonymous hemianopsia, and referred him to a neurologist to evaluate for cortical disease. His scores on cognitive tests indicated relatively preserved verbal memory and spoken language skills, with severely impaired visual perceptual skills measured by a Cortical

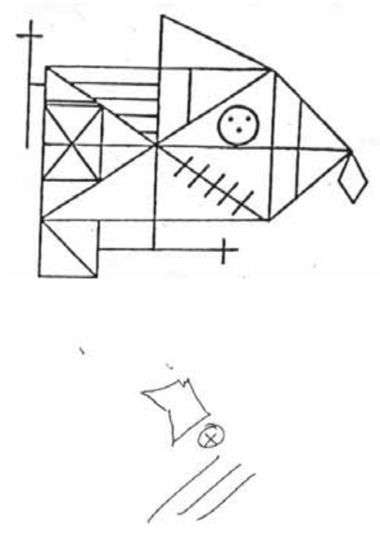

Fig. 3. Impaired Copying Ability. Fifty-eight year-old man with impaired visual-spatial skills due to PCA was unable to directly copy the Rey-Osterreith complex figure.

Vision Screening Test (43% error) and impaired copying measured by Rey-Osterreith Complex Figure (1 of 36 parts copied correctly) (Fig. 3). MRI demonstrated atrophy out of proportion to his age, most markedly in the right occipito-parietal region. He maintained independence in activities of daily living (except driving and financial affairs) over the next three years with the assistance of occupational therapy and specialists in low vision. Verbal language skills remained intact.

### *4.2. Case 2*

A 58 year-old artist was evaluated following a 7-year decline in visual perception. She had first noticed significant problems with reading, writing, calculations, and ability to handle money. Her husband noted that the poor visual perception affected many aspects of her daily activities, from difficulties assembling items such as an electric toothbrush, to problems dialing a telephone or finding the keys on a computer keyboard. Even her paintings were markedly affected, such that her style changed from realistic paintings to abstract paintings (Fig. 4). Cognitive testing revealed markedly impaired visual perception (Cortical Vision Screening Test, 20% errors), impaired constructional skills (Rey-Osterreith Complex Figure score of 17.5/36), and mild memory problems. Her MRI was notable for occipito-parietal atrophy, particularly on the left (Fig. 1, Panel B).

### *4.3. Case 3*

A 64 year-old man first noticed difficulties with his vision during his work as a bricklayer. He was having problems putting the bricks in line. Very soon afterward he noticed problems in reading and memory. He noted that he was able to see letters, but was unable to make sense of them. Also, when looking at a plate of food, he would see only one item on the plate.

Cognitive testing demonstrated marked deficits in visual perception (Cortical Vision Screening Test Score of 61/100), visual recognition and picture naming, along with milder deficits in memory and attention. When shown a bed, he named it, “clothespin” and then “teacher’s desk.” Consistent with simultanagnosia (a component of Balint’s syndrome), his description of a complex picture was limited to only a small portion of it. Similarly, examination of his reading revealed that he could read single isolated words, but had difficulty reading words when they were combined into sentences. There was also impairment in his visually guided reaching (another component of Balint’s syndrome). His MRI demonstrated atrophy in the parietal-occipital regions (Fig. 1, Panel C). His ophthalmologic examination was unremarkable.

## 5. Discussion

The presentation of PCA in this study of patients who were evaluated three to four years after onset of symptoms is consistent with previous case reports of the disorder, including symptoms of vision difficulty, alexia, acalculia, agraphia, anomia, constructional apraxia, and optic ataxia. Memory loss was also a commonly reported symptom of this group, but was significantly less common and severe than in the tAD group. Patients with PCA can progress to global dementia with memory loss becoming a prominent symptom as in tAD [2,4,9,10,27], but generally the decline in memory is slower and less severe. The large proportion of patients experiencing some memory loss in our study may be due to the relatively long disease duration. Although initial symptoms of PCA and tAD differ by definition, this study confirmed that these clinical syndromes can still be distinguished even three to four years after onset. The patients in this study had all been previously evaluated by a physician, and had been previously diagnosed with stroke (most commonly in patients whose initial symptom was visual field defect and/or dyslexia), Creutzfeldt-Jakob disease, or AD.

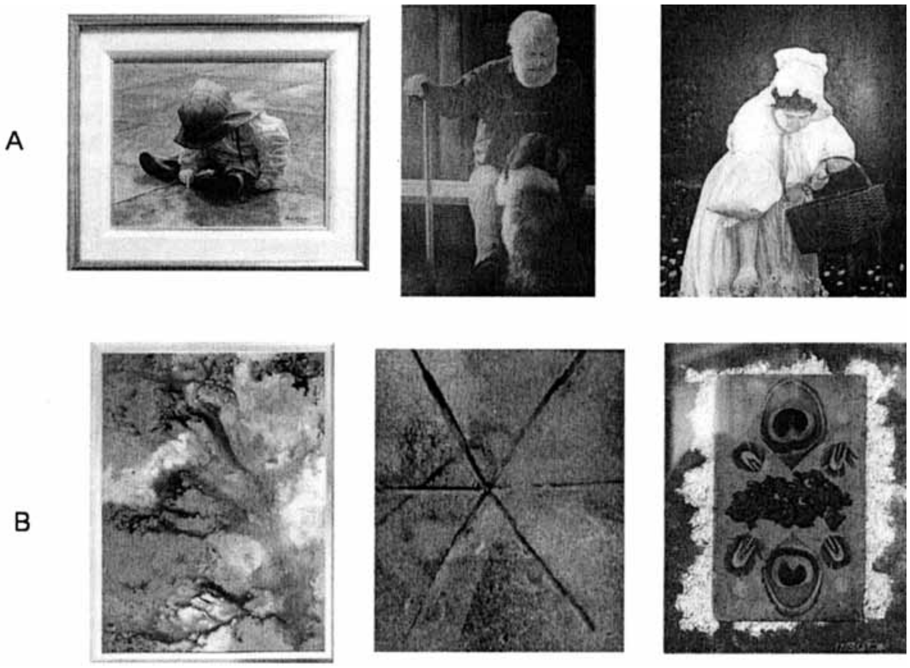

Fig. 4. Example of Visual Perceptual Changes in PCA. The paintings of a 58 year-old artist who developed PCA. Panel A. Near photographic renditions of the subject matter before onset of PCA; Panel B. Equally artistic but abstract paintings after onset of PCA.

The patients or their caregivers had in each case questioned the diagnosis, given the atypical symptoms or course of the disease. The diagnosis of PCA allowed the families to understand the atypical features and to make contact with other families of individuals with similar symptoms. The diagnosis of PCA also led to different predictions about the future course of the disease. Memory and insight rarely become as impaired as in tAD, making the prognosis more favorable in terms of retaining recognition of family and friends and in terms of recalling one's own history.

The goal of this study was also to identify quick neuropsychological tests that are practical to administer in the provider's office and that reliably distinguish PCA from tAD even a few years after onset of symptoms. Tests primarily evaluating bilateral occipital and parietal functions – visual perception, spatial attention, copying, reading – were evaluated as potential candidates. Results demonstrated that accuracy lower than 88% on the Cortical Vision Screening Test and a score lower than 18 on the Rey-Osterreith Complex Figure direct copying test had a high (100%) sensitivity and specificity for PCA. Because there was a high pre-test probability of PCA in this study, these cut-off scores need to be evaluated in a larger study with a population more representative of the range and proportions of various dementias seen in primary practice. Nevertheless, our results indicate that specific cut-off scores on a limited number of tests can distinguish these groups in approximately the same way as a whole battery of neuropsychological tests and imaging.

Both PCA and tAD cases demonstrated impairment in verbal recall as measured by the Rey Auditory Verbal Learning Test. However, delayed verbal recall was less impaired in patients with PCA than in tAD cases ($p = 0.030$). Therefore the presence of unimpaired delayed verbal recall is a distinguishing factor in diagnosing PCA. The difference in overall memory function in our study was not as marked as in previous studies, perhaps due to the relatively long disease duration in our PCA group. However, some of our patients with PCA (but no patients with tAD) did have preserved performance on the Rey Auditory Verbal Learning Test.

On the Boston Naming Test, both tAD and PCA cases showed impaired naming ability. The reason for the impairment is likely different in the two groups, however. Impaired visual recognition might account for the impaired naming in PCA, while anomia might account for the impairment in tAD. This hypothesis was supported by the distribution of error types in the two groups – a higher proportion of visual errors and visual-semantic errors in PCA cases and a higher proportion of circumlocutions and phonological errors in AD cases.

A limitation of this investigation was that the PCA patients were selected for their visual dysfunction, as well as imaging evidence of occipito-parietal atrophy, that together distinguished them from the tAD group at the time of diagnosis. Therefore, it is not surprising that the groups differed on tests of visual function. However, the goal of the study was to inform and educate the clinician on this uncommon dementing syndrome, and to identify neuropsychological tests that would help the clinician identify PCA (even without neuroimaging) or distinguish this clinical syndrome even relatively late in the course. Another limitation was the small number of patients studied; however, due to the rarity of this disorder, this is one of the larger studies investigating this illness. Finally, neuropathological results to determine the etiology of the clinical syndromes are not yet available.

Recognition of PCA, based on symptoms or tests that distinguish PCA from tAD, is crucial for prognosis and best management. Recognition of the existence of PCA allows the clinician to avoid incorrect diagnoses such as stroke. Although the most common etiology of PCA identified at autopsy is pathologically-defined Alzheimer's Disease, the course and clinical features of PCA are distinct from tAD even a few years after onset. Furthermore, prognosis for maintaining independence in most daily activities, maintaining recognition of familiar people, and maintaining recall of events is generally better in PCA than in tAD (except in cases of PCA due to Creutzfeldt-Jakob Disease, since this disease spreads quickly to the rest of the brain). Because the pathology is often the same in PCA and tAD, patients are often appropriately treated with acetylcholine esterase inhibitors that are approved for AD. The effectiveness of these medications in PCA is unknown. However, in addition to medications, patients with PCA often benefit from occupational therapy services, and/or referral to a low vision center, to assist in compensating for their visual perceptual deficits. Their relatively intact insight and learning ability favor a positive response to rehabilitation. Additionally, patients with PCA often enjoy books on tape, since they can follow and retain the plot. Finally, referral for a driving evaluation, or discontinuation of driving, is essential early in the course of PCA. The diagnosis of PCA allows formation of support groups of patients with PCA and their caregivers, who may have little in common with patients with tAD and their caregivers.

PCA is an uncommon dementing syndrome with characteristic deterioration of visual perception and other occipito-parietal functions, and relatively preserved verbal memory. Like different forms of FTLD, progressive supranuclear palsy, and corticobasal degeneration (which can have the same histopathology, but in different areas of the brain [36]), PCA and tAD can have the same histopathology but with different distributions. This study has demonstrated that symptoms (even a few years after onset), as well as performance on two quick visual-perceptual and copying tests can reliably distinguish PCA from tAD. The provider's awareness of this distinct clinical syndrome can lead to earlier identification, appropriate therapy, and more accurate prognosis for these patients.
